# The Impact of Motivational Interviewing and MOTIVE Tool Use by Pharmacists on Vaccine Acceptance

**DOI:** 10.3390/pharmacy12040114

**Published:** 2024-07-24

**Authors:** Aleda M. H. Chen, Alea Anthony, Adeola Balogun, Ruth Pereira, Justin W. Cole

**Affiliations:** School of Pharmacy, Cedarville University, 251 N. Main St., Cedarville, OH 45314, USA; aleaanthony@cedarville.edu (A.A.); abalogun@cedarville.edu (A.B.); rpereira@cedarville.edu (R.P.); jwcole@cedarville.edu (J.W.C.)

**Keywords:** vaccine acceptance, vaccine hesitancy, patient-centered communication, motivational interviewing, community pharmacy

## Abstract

Vaccines have played a significant role in reducing infectious disease burden. However, vaccine hesitancy remains a persistent challenge in public health, including for pharmacists who often interact with patients regarding vaccines. Thus, this study assesses the impact of motivational interviewing (MI) training and the MI-based vaccine hesitancy discussion tools (MOTIVE) on pharmacists’ management of vaccine hesitancy. Pharmacists in eight Midwestern pharmacy practices who completed MI and MOTIVE training and engaged with vaccine-hesitant patients participated in this study. The pharmacist participants completed post-encounter surveys identifying the vaccine discussed, the tool utilized, and the outcome of the conversation. Descriptive results from 362 encounters indicated that the primary reasons for hesitancy were safety (39%), care coordination (31.5%), and efficacy (30.4%). Post encounter, 35.4% of patients received vaccines, 26% planned to, 25.1% considered it, and 13.5% were uninterested. The findings highlight the importance of patient-centered communication, such as MI, between patients and pharmacists to identify and address reasons for vaccine hesitancy. Pharmacists, equipped with conversation tools such as the MOTIVE tools, may effectively influence vaccine acceptance. Future research should evaluate the utility of MI and the MOTIVE tools in other settings and regions.

## 1. Introduction

In the last century, vaccines have been a cornerstone of public health, leading to a substantial reduction in both cases and deaths from various infections such as smallpox, tetanus, diphtheria, pertussis, and mumps [1,2]. Presently, there are over 20 vaccines available for both adults and children, offering a preventive measure against infection-related morbidity and mortality [3]. The emergence of COVID-19 in 2019 intensified the focus on vaccination efforts globally, with the virus causing widespread illness, mortality, and societal disruptions [4,5].

Despite available vaccines to treat many diseases, vaccine hesitancy, or the reluctance or delay in accepting vaccines, [6] has risen in recent years. For example, many patients expressed hesitancy toward COVID-19 vaccines once they became available [7]. Current adult vaccination rates for other diseases remain suboptimal, especially for annual vaccines like influenza with a 48.6% coverage rate within the U.S., posing a challenge to public health goals outlined in Healthy People 2030 [8,9]. Vaccine hesitancy is deeply rooted in health beliefs, shaped by cultural, social, religious, and political influences [10]. The Health Belief Model provides a lens for understanding the relationship between health beliefs and vaccine hesitancy, considering factors such as perceptions of disease risk and severity as well as the perceived benefits of vaccination. Outside influences, including friends, family, the media, and social media, further impact health beliefs [10]. Addressing vaccine hesitancy and health beliefs requires an evidence-driven, patient-centered approach, emphasizing the importance of accurate information delivered in a collaborative and supportive manner.

Motivational Interviewing (MI) can be an effective approach to elicit information regarding patient health beliefs and move patients toward decisions [11]. It allows the healthcare provider to fully explore a patient’s concerns or knowledge gaps using a patient-centered approach and direct, open conversations [11]. Studies have explored the use of MI in vaccine conversations and found it to be effective in improving vaccine acceptance [12,13,14]. However, wide-scale implementation of MI has been limited, particularly in settings with rising vaccine delivery, such as community pharmacies. Given persistently low vaccination rates, especially for influenza, and the need to enhance vaccine confidence to achieve broader community immunity, it is important to provide the resources needed for healthcare providers to address vaccine hesitancy.

A structured tool based on MI principles and designed to be used with MI-based communication may be a useful resource for healthcare providers. One such tool is the MOTIVE (Motivational Interviewing Tool to Improve Vaccine Acceptance) tool, designed to help healthcare providers identify and address health beliefs contributing to vaccine hesitancy. The MOTIVE tool, in conjunction with provider MI training, has previously been used to address vaccine hesitancy in the ambulatory care setting [14]. Pharmacists are uniquely positioned in community and ambulatory care settings to promote vaccine confidence, particularly given that pharmacists are a low-hesitancy healthcare profession [15]. Community pharmacies are located in urban, suburban, and rural settings, with 90% of Americans having access to a community pharmacy within 5 miles of their home [16]. This is especially important as many patients are in medically underserved areas without convenient access to medical offices. Furthermore, pharmacists are increasingly incorporated into ambulatory care clinics [17] and can reach additional patients. Given the role of community and ambulatory care pharmacists in delivering vaccinations [18], the objective of this project was to determine the impact of pharmacist training and the use of the MOTIVE tools on patient vaccine hesitancy and vaccine acceptance.

## 2. Materials and Methods

Institutional Review Board approval (IRB #E089) was obtained from Cedarville University. 

### 2.1. Development of the MOTIVE Tools and Online Training Modules

In the first phase of this project, the prior MOTIVE tools [14] were updated. In brief, the original tools were developed for providers to use with parents or caregivers of pediatric patients (ages 0–5) who express hesitancy with the core pediatric immunization series (MOTIVE) or the seasonal influenza vaccine (MOTIVE-Flu). The MOTIVE tool was structured to provide a flow for a vaccine conversation using MI principles. The tools began with a presumptive statement regarding vaccination (ex: “Today, your child will be receiving…”), which matched guidance from the American Academy of Pediatrics at the time [19]. Then, open-ended questions were listed to elicit common health beliefs if a parent expressed vaccine hesitancy. Depending on the response, evidence-based information was listed in the tool to support the provider in addressing the beliefs. 

After updating the original tools to reflect best evidence, the tools were expanded to include additional vaccines: MOTIVE-Flu (Adult), MOTIVE-COVID (Adult and Pediatric), and MOTIVE-Shingles. The tools were also tailored for use in all age groups, including adults. For all tools other than the original MOTIVE (pediatric core immunizations), a participatory approach (ex: “What are your thoughts on receiving the vaccine today?”) was utilized in the tool, which matched newer guidance [20].

A series of four training modules were created to give providers information needed to implement the MOTIVE tools and have vaccine conversations. In our prior work, live educational modules were delivered covering MI principles, vaccine-preventable diseases, corresponding health beliefs, and the application of MI using the MOTIVE tools [14]. Given less access to in-person training due to the COVID-19 pandemic, the training was modified to online, self-paced learning containing four 1-h continuing education-accredited modules addressing: (1) an overview of MI, (2) vaccine hesitancy and health beliefs, (3) an overview of the MOTIVE tools, and (4) patient case studies in which the pharmacists need to apply MI skills and the tool. Multiple-choice questions were created and used for assessment purposes. After COVID restrictions lessened, the training modules remained online to allow for self-paced learning given the busyness of community pharmacies and preferences of the participants.

### 2.2. Site Recruitment and Training

Two types of pharmacy sites were recruited through state-level professional pharmacy organizations, school preceptor networks, and alumni databases in Ohio, Indiana, and Pennsylvania, with a goal of recruiting sufficient pharmacy locations to ensure that pharmacists would use the tool(s) in at least 300 patient encounters. 

Once recruited, any pharmacy staff member who interacted with patients about vaccines (pharmacists, pharmacy interns, and pharmacy technicians) at the sites completed a training series. The pharmacist(s) assumed primary responsibility for the intervention and documentation. A trained student research assistant followed up with each site to ensure that training was completed and then met with each site to (1) answer questions and (2) deliver the materials for the intervention.

Monthly educational reinforcement (approximately 5–15 min in length) was recorded and distributed to sites to ensure that the pharmacies had the latest vaccine information. MOTIVE tools were regularly reviewed, updated, and distributed to pharmacies to ensure that the evidence-based information was accurate. A trained research assistant then followed up every 2–3 weeks to determine how the intervention was going, if personnel at the site had sufficient pages left in the notebook (see the Data Collection section), and if there were any questions related to the intervention.

### 2.3. Data Collection and Analysis

Data were collected after each in-person encounter in which a MOTIVE tool(s) was used. All pharmacists interacted with patients in person when they came to the pharmacy. The pharmacy site documented the outcomes based on patient responses in the encounter. Items collected included: patient demographic information (e.g., age, race, and gender), reasons for hesitancy, vaccine addressed, patient post-encounter intent to receive the vaccine, and any continued hesitancy. The list of items collected was kept brief to allow the pharmacies to quickly record encounters without adversely impacting the workflow. All data was ultimately collected and housed in REDCap, a HIPAA-compliant data collection tool for research. Site-level data for community pharmacies were also obtained and included: site characteristics (e.g., hours open per week, pharmacist hours per week, and staff hours per week) and vaccines administered during the study period.

Some sites had the capacity to utilize all study materials electronically, while other pharmacies and settings did not have easy access to electronic resources outside of electronic health records. Thus, to maximize ease of integration into the setting, all study tools were made available in both paper and electronic formats. Once a site completed the training, during their onboarding by a trained research assistant, site personnel received a 5.5 × 8.5 inch spiral bound notebook that contained the following items: a QR code that hyperlinks to the data collection tool in REDCap, a QR code that hyperlinks to the MOTIVE tools in electronic format, and 100 data collection pages for post patient encounter (if paper was preferred over the REDCap entry). A notebook with the MOTIVE tools also was given to the pharmacy for reference.

An SPSS dataset was obtained from REDCap and imported for analysis. SPSS v. 28.0 (Armonk, NY, USA) was used to provide descriptive statistics.

## 3. Results

A total of 362 in-person patient encounters across eight community pharmacy and ambulatory care sites with patients expressing vaccine hesitancy were recorded between 11 October 2021 and 14 October 2023. Pharmacy information is presented in Table 1, and patient demographic information is presented in Table 2. 

Almost half of the encounters included patients over the age of 65 years (N = 178, 49.2%), and 88.7% (N = 321) of patients were White. The most frequently addressed vaccine was shingles (N = 178, 49.2%) followed by COVID-19 (N = 163, 45.0%) and influenza (N = 126, 34.8%) (Table 3 and Table 4).

Common reasons for hesitancy included safety (N = 141, 39.0%), coordination of care (N = 114, 31.5%), and efficacy (N = 110, 30.4%) (Table 4). After the encounter, 61.0% (N = 222) of patients either received the vaccine (N = 128, 35.4%) or prepared or planned to receive the vaccine (N = 94, 26.0%) (Table 5). The remaining patients shared that they may consider receiving the vaccine in the future (N = 91, 25.1%) or that they were not interested in the vaccine (N = 49, 13.5%) (Table 5). Among those who did not receive or plan to receive the vaccine, most patients reported that they were still hesitant (N = 74, 20.4%) or requested more time to process (N = 32, 8.8%); additional reasons for continued hesitancy are reported in Table 5.

## 4. Discussion

Vaccine hesitancy is often multifactorial and is influenced by a variety of internal and external factors. Whether related to personal experiences with vaccinations, political positions, or previous interactions in the healthcare system, patients and parents often present with multiple barriers to vaccinations for themselves or their children, respectively [21]. In a systematic review, parental attitudes and beliefs on vaccination were analyzed and seven key themes within vaccine hesitancy emerged [22]. The most frequently represented themes included “adverse effects,” (ex: vaccines may cause disease/illness or vaccines may overload the immune system) “mistrust,” and “lack of necessity” (ex: role of natural immunity or remedies) [22]. Another systematic review examined influenza and COVID-19 hesitancy and found safety, lack of trust, lack of need, and cultural reasons to be key factors in patient hesitancy along with the belief in the role of natural immunity [23]. Both of these studies as well as our prior work [14] have continued to find that these are key concerns among all patient groups. In the present study, safety was the most common reason for hesitancy (N = 141, 39.0%) followed by coordination of care (N = 114, 31.5%) and efficacy (N = 110, 30.4%). These are the larger health belief categories that subsume most of these concerns.

It is essential for healthcare professionals across settings and disciplines to address misconceptions and misinformation that can arise from a variety of sources [24,25]. This study found a high degree of vaccine acceptance, with 61.0% (N = 222) of patients either receiving the vaccine or planning to receive the vaccine in the future after having a conversation with the pharmacist. Prior reviews have found low to moderate evidence for the benefit of in-person vaccination conversations with parents, particularly among patients who have a lack of understanding about vaccine availability [26]. Other studies in community pharmacies that have examined MI-based in-person and telephonic interactions have found significant increases in vaccination rates: the rate increased for four of the five evaluated vaccines by 7–45% [27]. Furthermore, provider follow-up after declining an HPV vaccine resulted in higher odds of secondary acceptance in a cross-sectional study [28]. Our prior work also found decreased vaccine refusal and increased Hib and influence vaccine coverage when MI and the MOTIVE tools were used by a variety of healthcare professionals in a federally qualified health center in comparison to a retrospective cohort that did not receive the intervention [14]. Thus, this underscores the importance of providing systematic training for healthcare professionals to address misconceptions and misinformation using approaches that are patient-centered, such as MI.

Using MI-based principles like developing discrepancy between goals and current behaviors, rolling with resistance, and expressing early empathy [11] may be effective in improving vaccine acceptance, with increased acceptance in our study as well as others [12,13,14,27,29]. In the PromoVaQ RCT by Gagneur and colleagues, mothers (N = 2695) in maternity wards in Quebec were randomized to an MI-based intervention or a control group in order to assess changes in vaccination intention for their infants. The intervention group had a statistically significant improvement in intention to vaccinate their infants compared to the control group [29]. As we have found in our study, by engaging with patients and exploring their underlying sources of hesitancy, pharmacists can positively impact vaccine uptake among patients and parents who are reluctant to vaccinate. 

As frontline healthcare providers, pharmacists are equipped to respond to these concerns from patients or parents by providing individualized, evidence-based care through vaccination services. However, since these conversations can be challenging considering the complex nature of vaccine hesitancy, training pharmacists to employ active listening and to share information appropriately and compassionately is vital. This study employed a virtual, asynchronous approach with continuing education credit that allowed the pharmacists to train when convenient and receive current information to provide patient care. Furthermore, familiarizing pharmacists with available resources can lead to positive patient outcomes during these encounters, such as increased vaccine acceptance.

Although pharmacist-led interventions can promote vaccine confidence, some patients will remain hesitant after vaccination conversations. In the present study, 38.7% (N = 140) of patients reported that they were still hesitant or needed more time to process their decision. Not all patients or parents must make the decision immediately to vaccinate—they have autonomy to make decisions. However, pharmacists should be seen as a nonjudgmental resource to aid in decision-making. Helpful approaches include asking each patient/parent for permission before sharing information, addressing items related to the social determinants of health, and permitting adequate time for them to make their decision [30,31]. By individualizing these encounters, pharmacists can meet the specific needs of patients and parents while respecting their health beliefs.

### Limitations

While this study expands upon our prior work and continues to demonstrate a positive impact of MI and the MOTIVE tools on vaccine acceptance, there are limitations that should be considered when interpreting the findings. This intervention was utilized only when a patient expressed vaccine hesitancy, which may introduce selection bias. Secondly, the intervention was implemented in the Midwestern region of the US at eight community and ambulatory sites. It is important to note that our findings are similar to other MI-based interventions in other regions and settings [12,27,29], which aids in verifying our findings. However, similar studies should be conducted in other geographic regions, especially those with more diverse populations. Additional demographic data could be obtained in these studies, such as educational attainment, which is known to influence vaccine confidence. Further studies evaluating MI-based communication and vaccine education tools are needed, particularly for vaccines not specifically covered in this intervention. Additionally, studies involving larger populations and additional healthcare professions are needed along with the utilization of validated instruments.

## 5. Conclusions

After engaging with an MI-trained pharmacist and the MOTIVE educational tools, over half of the patients involved in the encounters either received a vaccine or planned to receive a vaccine. These results support the importance of in-person interactions with patients and parents to improve vaccine literacy and uptake. Equipping pharmacists for conversations with tools such as the MOTIVE tools may enhance conversations with patients and effectively influence vaccine acceptance. 

## Figures and Tables

**Table 1:** Site characteristics (N = 8).

Characteristic	Median (IQR)
Hours Open Per Week	49.0 (12.0)
Pharmacist Hours Per Week	69.0 (41.3)
Support Staff Hours Per Week ^1^	108.0 (140.3)

^1^ Support staff included pharmacy technicians and student pharmacists.

**Table 2:** Demographic information of patients expressing vaccine hesitancy (N = 362).

Demographic Parameter	N (%)
Age	
18–29	20 (5.5)
30–49	42 (11.6)
50–65	122 (33.7)
>65	178 (49.2)
Race	
Asian	1 (0.3)
Black/African American	2 (0.5)
White	321 (88.7)
Unknown/Not Reported	38 (10.5)
Gender	
Female	203 (56.1)
Male	159 (43.9)

**Table 3:** Total vaccinations administered during the study period.

Site ^1^	Total N	Per Month Median (IQR) ^2^
Site 1	3576	58.0 (235.5)
Site 2	741	13.0 (30.8)
Site 3	1188	39.0 (36.5)
Site 4	1409	57.5 (113.3)
Site 5	904	25.0 (96.3)
Site 6	291	8.5 (17.3)
Site 7	1715	62.5 (89.0)
Total	9824	39.0 (130.9)

^1^ Pharmacists at site 8 did not directly administer vaccinations in the ambulatory care clinic. ^2^ Sites had varying data collection periods ranging from 8 to 24 months.

**Table 4:** Reasons for hesitancy and vaccine(s) addressed with patients expressing initial hesitancy (N = 362).

Reason or Vaccine ^1^	N (%)
Reason for Hesitancy	
Safety	141 (39.0)
Efficacy	110 (30.4)
Ethical/Religious	3 (0.8)
Coordination of Care	114 (31.5)
Vaccine Addressed	
Influenza	126 (34.8)
COVID-19	163 (45.0)
Shingles	178 (49.2)
Other Vaccines ^2^	140 (38.7)

^1^ These were “select all that apply” questions. ^2^ Other vaccines addressed included: Tdap, pneumonia, hepatitis A, and hepatitis B.

**Table 5:** Patient post-encounter intent to receive the vaccine and reasons for continued hesitancy.

Intent or Reason (N = 362)	N (%)
Post-Encounter Vaccine Action/Intent	
Received a Vaccine	128 (35.4)
Preparing/Planning to Receive a Vaccine	94 (26.0)
Might Consider in the Future	91 (25.1)
Not Interested	49 (13.5)
Reasons for Not Receiving a Vaccine Today	
Not Enough Information	3 (0.8)
Still Hesitant	74 (20.4)
Needs More Information	7 (1.9)
Needs More Time to Process	32 (8.8)
Unknown	14 (3.9)

## Data Availability

Restrictions apply to the availability of these data.

## References

[1] Roush S.W., Murphy T.V., The Vaccine-Preventable Disease Table Working Group (2007). Historical comparisons of morbidity and mortality for vaccine-preventable diseases in the United States. J. Am. Med. Assoc..

[2] Henderson D.A. (2011). The eradication of smallpox—An overview of the past, present, and future. Vaccine.

[3] World Health Organization (2021). Module 1: Introduction to Vaccine Safety—History of Vaccine Development. https://vaccine-safety-training.org/history-of-vaccine-development.html.

[4] (2023). Johns Hopkins University of Medicine: Coronavirus Resource Center. COVID-19 Dashboard. https://coronavirus.jhu.edu/map.html.

[5] Centers for Disease Control (2024). Estimated Disease Burden of COVID-19. https://www.cdc.gov/coronavirus/2019-ncov/cases-updates/burden.html.

[6] MacDonald N.E. (2015). Vaccine hesitancy: Definition, scope and determinants. Vaccine.

[7] Pourrazavi S., Fathifar Z., Sharma M., Allahverdipour H. (2023). COVID-19 vaccine hesitancy: A Systematic review of cognitive determinants. Health Promot. Perspect..

[8] U.S. Department of Health and Human Services (2020). Healthy People 2030: Vaccination. https://health.gov/healthypeople/objectives-and-data/browse-objectives/vaccination.

[9] Centers for Disease Control and Prevention (2024). VaxView Vaccination Coverage. https://www.cdc.gov/vaccines/vaxview/index.html.

[10] Mercadante A.R., Law A.V. (2021). Will they, or Won’t they? Examining patients’ vaccine intention for flu and COVID-19 using the Health Belief Model. Res. Soc. Adm. Pharm..

[11] Miller W.R., Rollnick S. (2013). Motivational Interviewing: Helping People Change.

[12] Gagneur A., Lemaitre T., Gosselin V., Farrands A., Carrier N., Petit G., Valiquette L., De Wals P. (2018). Promoting Vaccination at Birth Using Motivational Interviewing Techniques Improves Vaccine Intention: The PromoVac Strategy. J. Infect. Dis. Ther..

[13] Lemaitre T., Carrier N., Farrands A., Gosselin V., Petit G., Gagneur A. (2019). Impact of a vaccination promotion intervention using motivational interview techniques on long-term vaccine coverage: The PromoVac strategy. Hum. Vaccines Immunother..

[14] Cole J.W., Chen A.M.H., McGuire K., Berman S., Gardner J., Teegala Y. (2022). Motivational interviewing and vaccine acceptance in children: The MOTIVE study. Vaccine.

[15] Barrett J. (2021). Survey: Majority of Pharmacists Confident in COVID-19 Vaccine. https://www.drugtopics.com/view/survey-majority-of-pharmacists-confident-in-covid-19-vaccine.

[16] American Society of Health-Systems Pharmacists (2020). Pharmacists as Front-Line Responders for COVID-19 Patient Care. https://www.ashp.org/-/media/assets/pharmacy-practice/resource-centers/Coronavirus/docs/Pharmacist-frontline-COVID19#:~:text=Pharmacists%20can%20help%20respond%20to,influenza%20and%20strep%20throat%20infections.

[17] Manolakis P.G., Skelton J.B. (2010). Pharmacists’ contributions to primary care in the United States collaborating to address unmet patient care needs: The emerging role for pharmacists to address the shortage of primary care providers. Am. J. Pharm. Educ..

[18] Deslandes R., Evans A., Baker S., Hodson K., Mantzourani E., Price K., Way C., Hughes L. (2020). Community pharmacists at the heart of public health: A longitudinal evaluation of the community pharmacy influenza vaccination service. Res. Soc. Adm. Pharm..

[19] American Academy of Pediatrics (2021). Talking with Vaccine Hesitant Parents. https://www.aap.org/en/patient-care/immunizations/communicating-with-families-and-promoting-vaccine-confidence/talking-with-vaccine-hesitant-parents.

[20] Berg S. (2021). 3 Tips to Tackle COVID-19 Vaccine Hesitancy among Black Patients. https://www.ama-assn.org/delivering-care/population-care/3-tips-tackle-covid-19-vaccine-hesitancy-among-black-patients.

[21] Cooper S., Schmidt B.M., Sambala E.Z., Swartz A., Colvin C.J., Leon N., Wiysonge C.S. (2021). Factors that influence parents’ and informal caregivers’ views and practices regarding routine childhood vaccination: A qualitative evidence synthesis. Cochrane Database Syst. Rev..

[22] Gidengil C., Chen C., Parker A.M., Nowak S., Matthews L. (2019). Beliefs around childhood vaccines in the United States: A systematic review. Vaccine.

[23] Kumar S., Shah Z., Garfield S. (2022). Causes of vaccine hesitancy in adults for the influenza and COVID-19 vaccines: A systematic literature review. Vaccines.

[24] Wood J.L., Eckert E., Von Isenburg M., Jackson J., Southwell B.G., Keselman A., Smith C.A., Wilson A.J. (2022). When medical practice meets medical myth: Confronting misinformation in the clinical encounter. Combating Online Health Misinformation: A Professional’s Guide to Helping the Public.

[25] Southwell B.G., Wood J.L., Navar A.M. (2020). Roles for Health Care Professionals in Addressing Patient-Held Misinformation Beyond Fact Correction. Am. J. Public Health.

[26] Kaufman J., Ryan R., Walsh L., Horey D., Leask J., Robinson P., Hill S. (2018). Face-to-face interventions for informing or educating parents about early childhood vaccination. Cochrane Database Syst. Rev..

[27] Coley K.C., Gessler C., McGivney M., Richardson R., DeJames J., Berenbrok L.A. (2020). Increasing adult vaccinations at a regional supermarket chain pharmacy: A multi-site demonstration project. Vaccine.

[28] Margolis M.A., Brewer N.T., Boynton M.H., Lafata J.E., Southwell B.G., Gilkey M.B. (2022). Provider response and follow-up to parental declination of HPV vaccination. Vaccine.

[29] Gagneur A., Battista M.-C., Boucher F.D., Tapiero B., Quach C., De Wals P., Lemaitre T., Farrands A., Boulianne N., Sauvageau C. (2019). Promoting vaccination in maternity wards—Motivational interview technique reduces hesitancy and enhances intention to vaccinate, results from a multicentre non-controlled pre- and post-intervention RCT-nested study, Quebec, March 2014 to February 2015. Eurosurveillance.

[30] Leask J., Kinnersley P., Jackson C., Cheater F., Bedford H., Rowles G. (2012). Communicating with parents about vaccination: A framework for health professionals. BMC Pediatr..

[31] Sangster A.V., Barratt J.M. (2021). Towards ending immunization inequity. Vaccines.

